# Malaria ecotypes: their usefulness for stratification in current malaria control and modeling

**DOI:** 10.1186/1475-2875-11-S1-P13

**Published:** 2012-10-15

**Authors:** Allan Schapira, Konstantina Boutsika

**Affiliations:** 1 Swiss Tropical and Public Health Institute, Socinstrasse 57, P.O. Box CH-4002 Basel, Switzerland; 2 University of Basel, Petersplatz 1 ,CH-4003 Basel, Switzerland

## 

To deal with the variability of malaria, control programmes need to stratify their malaria problem into a number of smaller units on the basis of the epidemiology of malaria or on determinants such as ecology. Relying on published research and grey literature we reviewed earlier classifications of malaria based on ecology. We found that all malaria in the world could be assigned to one or more of the following ecotypes: savanna, plains and valleys; forest and forest-fringe; foothill; mountain-fringe and northern and southern fringes; desert-fringe; coastal and; urban. Such classification provides a framework for planning, when it is recognized that the implications of any ecotype depend on the biogeographical region, sometimes sub-region, and that knowledge on physiography must be supplemented by information on natural, anthropic and health system processes. Only two ecotypes can be delimited with some accuracy and have relatively constant implications for control within certain biogeographic regions: forest environments in the Indo-malay and the Neo-tropic and urban malaria, which has different implications in Africa and in the Indian sub-continent.

